# Technological Advances of 3D Scaffold-Based Stem Cell/Exosome Therapy in Tissues and Organs

**DOI:** 10.3389/fcell.2021.709204

**Published:** 2021-09-09

**Authors:** Chenyang Gu, Jia Feng, Ahmed Waqas, Yushu Deng, Yifan Zhang, Wanghao Chen, Jun Long, Shiying Huang, Lukui Chen

**Affiliations:** ^1^Department of Neurosurgery, Neuroscience Center, Integrated Hospital of Traditional Chinese Medicine, Southern Medical University, Guangzhou, China; ^2^School of Medicine, Southeast University, Nanjing, China; ^3^Department of Neurosurgery, Ninth People Hospital Affiliated to Shanghai Jiao Tong University School of Medicine, Shanghai, China; ^4^School of Traditional Chinese Medicine, Southern Medical University, Guangzhou, China

**Keywords:** 3D bioprinting, scaffold, regenerative engineering, stem cell, exosome, therapy

## Abstract

Recently, biomaterial scaffolds have been widely applied in the field of tissue engineering and regenerative medicine. Due to different production methods, unique types of three-dimensional (3D) scaffolds can be fabricated to meet the structural characteristics of tissues and organs, and provide suitable 3D microenvironments. The therapeutic effects of stem cell (SC) therapy in tissues and organs are considerable and have attracted the attention of academic researchers worldwide. However, due to the limitations and challenges of SC therapy, exosome therapy can be used for basic research and clinical translation. The review briefly introduces the materials (nature or polymer), shapes (hydrogels, particles and porous solids) and fabrication methods (crosslinking or bioprinting) of 3D scaffolds, and describes the recent progress in SC/exosome therapy with 3D scaffolds over the past 5 years (2016–2020). Normal SC/exosome therapy can improve the structure and function of diseased and damaged tissues and organs. In addition, 3D scaffold-based SC/exosome therapy can significantly improve the structure and function cardiac and neural tissues for the treatment of various refractory diseases. Besides, exosome therapy has the same therapeutic effects as SC therapy but without the disadvantages. Hence, 3D scaffold therapy provides an alternative strategy for treatment of refractory and incurable diseases and has entered a transformation period from basic research into clinical translation as a viable therapeutic option in the future.

## Graphical Abstract

A summary schematic of application of 3D scaffold-based stem cell/exosome therapy.

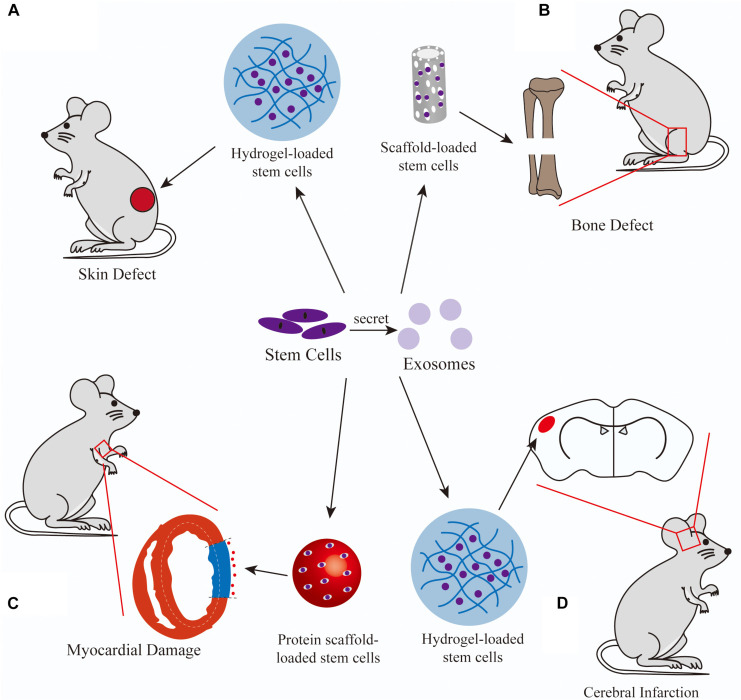

## Introduction

The self-renewal capacity of human cells decreases with age and disease. Regenerative engineering of complex tissue structures is a relatively recent discipline that combines materials research, mechanics, stem cell (SC) science, and clinical translation. New therapies for regeneration of weakened tissues and organs have focused on the use of autographs and tissues from living and deceased donors. However, these therapies are dependent on the availability of donor tissues and site morbidity. Hence, the field of tissue engineering and regenerative medicine has been rapidly developing to meet the need of biological substitutes.

Stem cells, present in the human embryo, fetus, umbilical cord blood, and adult (Ferraro et al., 2016), possess the ability to self-renew in an undifferentiated state in culture, while retaining the ability to differentiate into specific cell types (Shi et al., 2018). SCs, which are usually in a quiescent state, help to maintain homeostasis of tissues and organs by preserving the progenitor characteristic via self-renewal. Stimulation with external factors and/or components of damaged host cells derived from the tissue microenvironment trigger the proliferation and differentiation of SCs (Shi et al., 2018).

**FIGURE 1:**
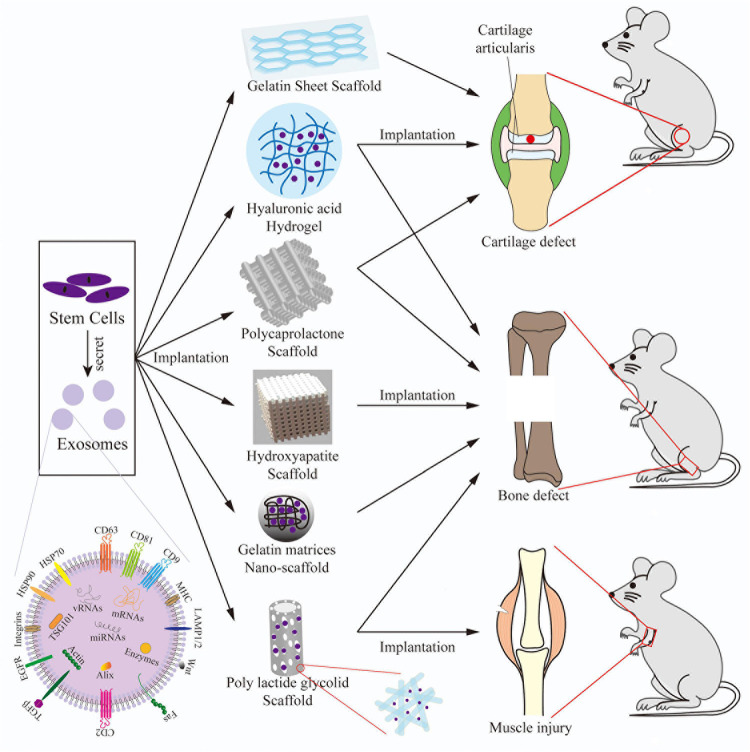
A schematic of the main types of 3D bioprinting. The application process of a 3D scaffold combined with SCs/exosomes implanted into bone diseases. A structural diagram of a representative exosome. SCs/exosomes are implanted in/on the 3D scaffolds, then the composite scaffolds are implanted into models of cartilage defects, bone defects, and muscle injuries.

Exosomes are extracellular vesicles produced by various eukaryotic cells that are much smaller than red blood cells, extracellular vesicles, microvesicles, and apoptotic bodies with diameters of 30–150 nm. Exosomes are either directly released from cells or by budding of the plasma membrane (Booth et al., 2006). The cargo of exosomes include multiple proteins, lipids, cytokines, RNA molecules, and chemokines (Simons and Raposo, 2009) (Figure 1). The activities of exosomes include immune control, mediation of cell proliferation, migration, division, and apoptosis, maintaining a physiological state, and participating in disease processes (Tkach and Théry, 2016). In addition, exosomes play various roles in the processes of coagulation, waste management, and intercellular signaling transduction (van der Pol et al., 2012). Therefore, the therapeutic application of exosomes has gained widespread popularity.

Over the past decade, the field of regenerative engineering has continued to evolve, and various functional biomaterials compatible with the human body have been developed for the treatment of diseased and damaged tissues (Riester et al., 2020). The inner structure of biomaterials can provide three-dimensional (3D) microenvironment that influence various aspects of cell behavior, many of which can have crucial physiological effects (Gattazzo et al., 2014). Indeed, biomaterials can be prepared with cells and cell-derived microvesicles, such as exosomes, from different sources to elicit a therapeutic effect in injured and diseased tissues. In addition, various 3D scaffold-based SC/exosome therapy have been developed for the treatment of diseased tissues and organs. Biomaterials include natural, biological and synthetic materials in various shapes and forms, such as injectable substances, gel, particles, and polymers.

## Materials and Methods

### Preparation of 3D Scaffold

#### Biomaterials

Biomaterials possess multiple attractive physical and chemical characteristics, including biocompatibility, printability, crosslinking capacity, biodegradability, specificity, controlled release, and bioplasticity (Williams, 2008; Bose et al., 2017). As carrier molecules, biomaterials also provide a specific 3D microenvironment to promote cell growth and function (Mitrousis et al., 2018; Wang et al., 2019). Biodegradability is considered to simulate the natural microenvironments of tissues and organs. Printability is the ability to temporally and spatially deposit biomaterials accurately. Crosslinking is a technique that uses a balancing system, printability, and bioactivity to rapidly mold soft printable materials into 3D structures to facilitate the preparation and printing of scaffolds. Specific formulations of various natural and synthetic biomaterials are vital to form successful 3D scaffold.

Natural materials include collagen (Chang et al., 2011), gelatin (Chang et al., 2020), fibrin (Rabbani et al., 2017), silk fibroin (Qing et al., 2018), alginate (Yang et al., 2018), gellan gum, hyaluronic acid (HA) (Yazdani et al., 2019), chitosan (Kim et al., 2016), Matrigel (Wang et al., 2020b); while synthetic polymer materials include poly caprolactone (PCL) (Zhang et al., 2017), polydimethylsiloxane (PDMS) (McKee et al., 2017), polyethylene oxide (PEO) (Evrova et al., 2016), polylactic acid (PLA) (Gandolfi et al., 2020), poly lactic acid-glycolic acid (PLGA) (Swanson et al., 2020), polyvinyl alcohol (PVA) (Kalachaveedu et al., 2020), polyethylene glycol (PEG), tribasic calcium phosphate (TCP) (Ying et al., 2020). Biomaterials used for 3D bioprinting are called “bio-inks.”

### Preparation and Crosslinking

To better adapt to functional tissues and organs, 3D scaffolds should be designed to promote cell proliferation and tissue regeneration. Hydrogels are attractive materials for cell encapsulation for transplantation of engineered cells and tissues. Natural biological materials, such as collagen, gelatin, fibrin, silk fibroin, alginate, and HA, are abundant in the body (Moshayedi and Carmichael, 2013). Hydrogel is a biocompatible and bioabsorbable material that allows the degradation of cells and matrices (Hanjaya-Putra et al., 2012), and can be modified and/or crosslinked by chemical modification to become more suitable for specific cells, tissues, and organs (Lam et al., 2014).

Scaffolds containing encapsulated particles avoid the integration of cells and degradation of drugs to exert a therapeutic effect similar to that of an infiltrative mini pump. To achieve a higher degree of cell attachment to a scaffold, various characteristics of particles should be considered to ensure that scaffolds adapt to the host-tissue architecture and can pass through a microinjector, which influences cell attachment, including materials, curvature, surface motif, electrostatic charge, and interactions with cells (Hejcl et al., 2008; Chen et al., 2010).

A porous scaffold provides a suitable matrix and environment for cell adhesion and growth, by seeding in the construct or allowing cells to proliferate, migrate, and differentiate, while interacting with surrounding tissues and organs (Park and Bronzino, 2002). Solid implantation scaffolds are usually created by computer-aided design and constructed by 3D bioprinting.

### Types of 3D-Bioprinting (Figure 1)

#### Extrusion-Based Bioprinting

Extrusion-based bioprinting (also called direct ink writing) is a type of 3D fabrication. Bio-inks are discharged from a syringe in continuous filaments that can be layered to create the desired design. The height of the architecture is based on piling several layers of these filaments. There are two types of extrusion-based bioprinting: (1) pneumatic extrusion bioprinting, which uses compressed air to push the bio-ink from a syringe (Lee et al., 2015b); and (2) mechanical extrusion bioprinting, which uses a stepping motor connected to a piston or screw to extrude the bio-ink. The mechanical outlet mechanism is more precise than the pneumatic system and can print semi-solid and solid bio-inks more effectively (Skardal et al., 2010; Fielding et al., 2012). Micro-extrusion is a form of extended extrusion bioprinting in which the diameter of the extrusion syringe is less than 1 mm (Murphy and Atala, 2014).

#### Droplet-Based Bioprinting

There are four types of droplet-based bioprinting. Inkjet-based bioprinting utilizes gravity, atmospheric pressure, and fluid mechanics to generate and eject liquid droplets onto a receiving matrix (Liu and Derby, 2019). However, the high pressure may be harmful to cells contained in droplets when ejected through a syringe with a very small diameter.

Electrohydrodynamic jet bioprinting, which utilizes an electric field to drag bio-ink droplets out the syringe, thereby eliminating the need for great pressure (Onses et al., 2015), is suitable for applications that require syringes with small diameters (≤100 μm) and high concentrations of bio-inks (Jayasinghe et al., 2006).

Acoustic droplet ejection bioprinting is an extension of DOD to pattern cells that uses the acoustic radiation force related to the ultrasonic field to shift momentum from the gas–liquid interface to the formation of droplets (Elrod et al., 1989). A specific amount of liquid is ejected when the sound pressure of the ultrasonic field is greater than the surface tension (Demirci and Montesano, 2007).

Laser-assisted bioprinting is a predetermined computer-aided design technology with the use of a scanning mirror system that is focused on the laser-induced forward transfer effect and laser-guided direct writing.

Photocuring-based bioprinting uses liquid light-curable resins in the photopolymerization phase that chemically react to create solid artifacts when exposed to light. Stereolithography (Melchels et al., 2010) and digital light processing (DLP) (Patel et al., 2017; Schmidt and Colombo, 2018) are both photopolymer additive manufacturing techniques.

## Stem Cells (SCs)

### Classification of Stem Cells

#### Embryonic Stem Cells (ESCs)

Embryonic stem cells (ESCs), upon separation from the inner mass of blastocysts, have stable developmental potential and the capacity of prolonged undifferentiated proliferation to form the three primary germ layers (i.e., the ectoderm, mesoderm, and endoderm) (Thomson et al., 1998).

#### Induced Pluripotent Stem Cells (iPSCs)

Most somatic cells can be reprogrammed to pluripotent SCs by cultivation *in vitro* for a few weeks with retrovirally imported transcription factors, such as c-Myc, Kfl4, Oct3/4, and Sox2. In addition, somatic cells reversed to the embryonic pluripotent state, similar to ESCs, can generate all the cell types of the body (Takahashi and Yamanaka, 2006; Takahashi et al., 2007). On account of this discovery, Dr. Shinya Yamanaka won the 2012 Nobel prize in Physiology or Medicine.

#### Mesenchymal Stem/Stromal Cells (MSCs)

Mesenchymal stem/stromal cells (MSCs), which are relatively easy to isolate and extensively expand, differentiate into various types of cells, mainly chondrocytes, osteoblasts, and adipocytes (Dominici et al., 2006), which exhibit adherent and fibroblast-like characteristics (Friedenstein, 1976). MSCs have been isolated from various tissues, including bone marrow (bone marrow-derived MSCs, BMSCs), adipose tissue (adipose-derived MSCs, ASCs), umbilical cord blood (umbilical cord-derived MSCs, UCMSCs), dental pulp, skeletal muscle, Wharton’s jelly, synovial membrane, and amniotic fluid (Keating, 2012).

#### Tissue Specific Stem Cells

Neural stem/progenitor cells (NSC/NPCs) are derived by separation from the adult and fetal brain. A satellite cell is a type of SC that is derived from skeletal muscle.

### Stem Cell Therapy

#### Bone

Stem cell treatment has progressed rapidly over the last two decades and it is now used as a tissue engineering technology in orthopedic surgery for the treatment of bone fractures, cartilage abnormalities, ligament-tendon injuries, and bone defects. For osteoarthritis (OA), Zhou et al. (2018) reported that local injection of ASCs into a rat model could alleviate histologically confirmed OA of the knee. Sasaki et al. (2017) reported that the enhanced proliferation could almost double the abundance of MSCs *in vitro*. Injection of MSCs cultured with granulocyte-colony stimulating factor was shown to induce regeneration of the hyaline cartilage for repair of trochlear osteochondral defects (Harada et al., 2015). In a clinical study of OA of the Knee, Al-Najar et al. (2017) reported that intraarticular injections of MSCs had significantly improved knee joint function.

#### Heart

Cardiovascular diseases remain the leading cause of death globally. Heart failure, which is characterized by avascular necrosis of cardiomyocytes at the terminal stage of disease, is caused by dilated cardiomyopathy, coronary heart disease, and severe valvular disease (Elgendy et al., 2019). However, since the efficacy of current treatments to reverse heart failure during myocardial infarction (MI) is limited, cardiomyocyte recovery has become a top priority. Various types of SCs, especially iPSCs, cardiac progenitor cells, and cardiosphere-derived cells, have been applied in myocardial repair. iPSCs have superior therapeutic efficacy in myocardial repair and iPSC-derived cardiomyocytes exhibit many of the same features as cardiac cells, including contractility, spontaneous pumping, and cytokine-mediated ion channel expression (Xi et al., 2010). With the use of a large animal model of immunosuppression-associated MI, Ishida et al. (2019) demonstrated that xenogeneic epicardial transplantation of iPSC-derived cardiomyocytes significantly improved cardiac function and resulted in a significant increase in capillary density in ischemic regions. Cell therapy trials (Tarui et al., 2015; Ishigami et al., 2017) revealed that intracoronary administration of cardiosphere-derived cells improved heart failure, somatic development, and quality of life by reverse remodeling.

#### Skin

For wound treatment, local injection of MSCs reduced apoptosis (Öksüz et al., 2013; Abbas et al., 2018) and improve burn wound progression (Öksüz et al., 2013) in animal models. Local treatment also improved survival after burn injury (Caliari-Oliveira et al., 2016). Furthermore, local injection of MSCs was shown to significantly accelerate the wound healing rate (Caliari-Oliveira et al., 2016; Abo-Elkheir et al., 2017; Chen et al., 2017), re-epithelization (Shi et al., 2017), granulation tissue formation (Caliari-Oliveira et al., 2016; Shi et al., 2017), and vascularization/angiogenesis (Caliari-Oliveira et al., 2016; Hosni Ahmed et al., 2017). SCs accelerated the wound healing process by inducing neo-angiogenesis (Atalay et al., 2014; Liu et al., 2014; Lough et al., 2014), collagen deposition (Liu et al., 2014) and granulation tissue formation, in addition to modulating the inflammatory response (Atalay et al., 2014; Liu et al., 2014; Caliari-Oliveira et al., 2016) and reducing the risk of infection. SC therapy was also shown to improve the healing of burn wounds and the immune response.

#### Eye

Boucherie et al. (2013) reported that transient neuroepithelium, which was generated by ESCs cultured together with a Matrigel extracellular matrix, had induced conversion into retinal progenitors in 5 days. The retinal progenitors had differentiated into Crx1-expressing photoreceptor precursors after just 10 days and then attained rod photoreceptor identity within 4 weeks. Huo et al. (2010) reported that most MSCs injected to the subretinal space of rats with retinosis remained in the cones and retinal pigment epithelium (RPE), and differentiated into retinal pigment epithelial and photoreceptor cells. Further, pan-cytokeratin and rhodopsin were expressed in engrafted MSCs. Tucker et al. (2011) reported that transplantation of iPSCs into immune-compromised mice with retinal degeneration formed teratomas containing all three germ layers and gradually exhibited normal retinal physiological characteristics in response to the delivery of neurotransmitters.

#### Nerve System

The SCs can restrict secondary injury, reduce inflammation, and secrete paracrine factors to protect surviving neurons. SCs also facilitate axon regeneration, and differentiate into new neurons to replace injured neurons in spinal cord injury (SCI) (Leng et al., 2019; Zhang et al., 2019; Feng et al., 2021). The results of a phase I clinical trial conducted by Mendonça et al. (Mendonça et al., 2014) showed that administration of BMSCs improved behavioral scores, electromyography, and somatosensory evoked potentials. A long-term follow-up study revealed that administration of BMSCs improved upper-limb motor capacity, electrophysiological function, and quality of life in three patients with grade B impairment (Park et al., 2012). Lower doses of ASCs were used to increase the cell migration rate and infarct volume of permanent occlusion in rat models of stroke during functional rehabilitation (Grudzenski et al., 2017).

### Scaffold-Based Stem Cell Therapy

#### Bone

Mesenchymal stem cells are often used in bone regeneration engineering. For cartilage formation: Xia et al. (2018) reported that pericellular coating with collagen I promoted the adhesion of MSCs to cartilage slices and direct intra-articular injection of MSCs enhanced homing and retention in cartilage defects. Intercellular associations were also stimulated by pericellular coating with collagen I, which promoted the expression of aggrecan, *N*-cadherin, and collagen II. The increased homing rate was related to intercellular contact. Sawatjui et al. (2015) reported that a silk fibroin-gelatin-chondroitin sulfate-HA scaffold outperformed a single silk fibroin scaffold *in vitro*. The hybrid scaffold might serve as a supporting system as well as a cartilage modeling environment for chondrogenesis by facilitating cartilage regeneration. This hybrid scaffold can potentially improve chondrogenesis by inducing proliferation and chondrogenic differentiation of MSCs. Chang et al. (2020) reported that honeycomb-like gelatin scaffolds can promote the survival of UCMSCs and chondrogenic differentiation to form hyaline-like cartilage. Zhang et al. (2017) reported that MSCs in a PCL scaffold improved fibrocartilage regeneration and mechanical efficiency, providing a practical alternative to shield the articular cartilage from injury following meniscectomy. Zorzi et al. (2015) reported that as compared to the control group, ASCs seeded on collagen/chitosan scaffolds improved the healing and thickness of chondral lesions in an adult ovine model.

Further clinical studies conducted by Gobbi et al. reported that an HA-based active bone marrow concentrate scaffold for management of small or significant lesions, one or multiple lesions in one or two compartments, and subsequent lesion therapy resulted in reasonably stable long-term results in cartilage repair (Gobbi and Whyte, 2016) and knee full-thickness cartilage injury (Gobbi and Whyte, 2019).

Moreover, Tseng et al. (2020) reported that the grafting of a layer of the anti-oxidative reagent *N*-acetylcysteine and extracellular matrix-like type I collagen increased the adhesion capabilities of rat ASCs and the proliferation of SCs *in vitro*. In addition, dynamic compression stimulus accelerated the differentiation of rat ASCs into the chondrogenic lineage within a relatively brief period.

Besides, McKee et al. (2017) reported that increased mRNA expression of RhoA in differentiated ESCs implanted into PDMS scaffolds as a result of compression-induced stress. When the ESCs in PDMS scaffolds were subjected to compression-induced stress and treated with an RhoA inhibitor, the chondro-inductive effect of RhoA was downregulated along with the transcription and translation of early markers of chondrogenesis.

##### Ossification

Yang et al. (2018) reported that peptide-modified porous alginate scaffolds enhanced the adhesion, proliferation, and aggregation of MSCs *in vitro*. The alginate polymers also exhibited complex bioactivity and functioned as an osteogenesis-promoting scaffold. Fu et al. (2018) stated that *in vitro*, layer-by-layer gelatin scaffolds modified with poly-L-lysine and minerals improved the adhesion, proliferation, and osteogenic differentiation of MSCs derived from dental pulp. *In vivo*, modified scaffolds promoted the formation of mineralized deposits and the expression of osteocalcin during osteogenic differentiation of MSCs. Ma et al. (2017) reported that incorporation of minced BMSC sheets into hydroxyapatite particles efficiently promoted bone formation *in vitro* by enhancing alkaline phosphatase activity and the mineralized area, while increasing angiogenesis, collagen deposition, and bone mass *in vivo*. Wang et al. (2015) reported that the combination of a decalcified bone matrix scaffold and BMSCs generated significantly more bone tissue in ovariectomized rabbits as confirmed by X-ray. However, osteoporosis adversely affected the treatment of defect and significantly reduced bone regeneration. To establish more reliable therapies for management of bone defects associated with osteoporotic disorders, the negative consequences of pathological factors should be carefully considered. Su et al. (2020) reported that the biocompatibility and bioactivity of nano-hydroxyapatite-chitosan-poly lactide-coglycolide scaffolds, which offered an appropriate microenvironment to prolong the replicative senescence of UCMSCs, thus maintaining stemness and youth, as compared to traditional long-term culture *in vitro*.

A clinical study conducted by Skoloudik et al. (2018) reported that the combination of BMSCs and hydroxyapatite scaffolds regenerated temporal bone defects and restored complete hearing to near preoperative levels. This approach could become an effective alternative for treatment of bone defects.

Moreover, a short-term follow-up study by Uri et al. (2018) reported that implantation of autogenous ASCs to the proximal femur of rats with osteoporosis associated with ovariectomy had directly transformed into osteoblasts and improved bone strength. Kroeze et al. (2015) reported that ASCs in combination with a poly-L-lactide-PCL scaffold had no negative effects, but did not increase the rate or quantity of fusions between antibodies under specified conditions. However, higher mineralized tissue content was observed in the autologous bone graft group.

##### Muscle healing

Chiu et al. (2020) reported that BMSC therapy accelerated the repair of skeletal muscle by improving rapid twitch and tetanus muscle strength after muscle contusion and increased the rate of myofiber regeneration. BMSCs combined with a Pluronic F-127 scaffold improved the function of contused muscles and promoted new muscle formation. Kheradmandi et al. (2016) reported that the physical and biological advantages of a porous and potent chitosan-polyvinyl-alcohol nanofibrous scaffold, leading to considerable viability, proliferation, and attachment of BMSCs in scaffolds implanted into the muscle tissue of rabbits, indicating significant cell-scaffold interactions and proliferation of major cells, with far less immunoreactivity.

Besides, Evrova et al. (2016) reported that a hybrid PLGA–PEO fibrous scaffold formed by blending of PEO to PLGA fibers supported the adhesion and proliferation of myoblasts, resulting in significant myotube formation and self-alignment, even if the scaffold was randomly oriented. The hybrid scaffold exhibited the strongest performance in terms of orientation, myotube shape, and mechanical properties, suggesting that the best biosynthetic microenvironment for myoblast segregation. The tuning fiber properties provided a valuable tool for engineering fibril microenvironments for several biomedical applications.

This section summarized the recent progress of SC therapy combined with 3D scaffolds for bone regeneration, cartilage formation, ossification, and muscle healing. Hence, 3D scaffolds based on gels, polysaccharides, or their derivatives combined with SCs can improve bone diseases (Figure 1).

#### Heart

Scaffold-based SC therapy with the use of MSCs, iPSCs, and ASCs has been applied for the regeneration of cardiomyocytes following MI.

Wang et al. (2020c) reported that injection with a collagen scaffold improved the durability of transplanted UCMSCs, implying greater angiogenesis and cardiomyocyte viability after MI.

Firoozi et al. (2020) confirmed cardiac-compatible properties of a self-assembling peptide (SAP) hydrogel with mild gelation, injectability, repair ability, and possible sequence alteration. An SAP hydrogel composite with a self-assembling gel-forming core sequence (RADA) was modified with a SDKP motif to facilitate pro-angiogenic and anti-fibrotic behaviors for use as a cardioprotective scaffold. With the addition of RADA-SDKP, injection of the compound hydrogel restored cardiac function following acute MI and gradually improved clinical outcomes.

Rabbani et al. (2017) reported that the combination of Wharton jelly-derived MSCs with a novel compound containing PEG, HA, and chitosan for heart regeneration. The cell/scaffold complex improved defect size and cardiac function, while promoting neoangiogenesis and cardiomyogenesis. Also, Rojas et al. (2015) reported that a fibrinogen biomatrix improved the retention of cardiac iPSCs and sustained improved cellular refill and function of infarcted myocardium. Therefore, fibrinogen could be considered as an ideal natural intramyocardial scaffold in the future.

Gelmi et al. (2016) reported that a conductive polymer polypyrrole-coated PLGA scaffold provided a microenvironment, including electrical and mechanical stimulation, which could promote proliferation, differentiation, and survival of transplanted SCs by controlling the microenvironment and mimicking the structural architecture of the heart. The hybrid scaffold is used to stimulate the viability and mediate the differentiation of iPSCs. Khan et al. (2015) stated that the morphology and roles of iPSC-derived cardiomyocytes cultured under an anisotropic atmosphere provided through an aligned nanofiber patch changed more than when cultured on a flat surface. These cells more closely resembled natural cardiac tissue and, thus, were suitable for cardiac implantation.

Besides, Bai et al. (2019) reported that an extracellular matrix (ECM) hydrogel greatly promoted the proliferation of SCs derived from brown adipose tissue and cardiomyogenic differentiation. The combination of SCs derived from brown adipose tissue and an ECM hydrogel retained cardiac activity and chamber geometry.

Oltolina et al. (2015) reported that spheroids were aggregated by seeding cardiac progenitor cells onto methylcellulose hydrogel-coated microwells. When spheroids were inserted into the heart walls and cardiotoxin-injured myocardium of female mice, the cells from the spheroids exhibited the very same engraftment capacity. Jamaiyar et al. (2017) reported that induced vascular progenitor cells (iVPCs) in a micro-bundle scaffold achieved greater engraftment, survival, and retention in the myocardium. Treatment with iVPCs and polymer micro-bundles enhanced the viability of cardiomyocytes, heart efficiency, and valve density, and reduced infarction size as determined by echocardiography.

The use of MSCs, iPSCs, and ASCs in combination with different types of 3D scaffolds achieved more significant effects than traditional therapies, while improving cardiomyocyte survival and improving infarcted tissues to varying degrees.

#### Skin

Kalachaveedu et al. (2020) reported that an electrospun nanofibrous Guar gum/PVA-based scaffold matrix, which incorporated four traditional medicinal plant extracts, was characterized by good water absorption and thermal stability. The scaffold outperformed skin in terms of elastic modulus, fiber spinnability, and tensile strength. Integrating a mat of herbal nanofibers and MSCs achieved complete skin repair with minimal scarring. Han et al. (2019) reported that activation of the Wnt signaling pathway promoted wound healing for diabetics by regulating the proliferation and differentiation of UCMSCs in a collagen-chitosan acellular dermal matrix scaffold. Shou et al. (2018) reported that the defensive use of a hydrogel composed of 3D chitin nanofibers increased the viability of BMSCs and acted as a functional scaffold that enhanced the regenerative capacity of BMSCs to facilitate wound healing.

Lee et al. (2020) reported that the ASC-derived spheroids were tightly entrapped by electrospun alginate nanofibers and alginate strut, which then released numerous factors related to angiogenesis and wound healing in a coordinated manner. The scaffold loaded with spheroids facilitated the formation of capillary-like structures in umbilical vein epithelial cells.

**FIGURE 2:**
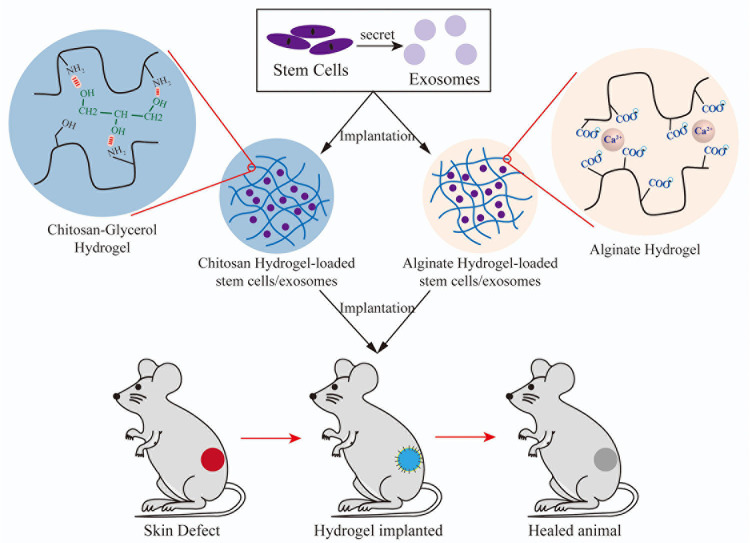
The process of implantation of different hydrogels combined with SCs/exosomes into models of skin defects and wound healing.

In summary, SC therapy, including MSCs and ASCs, combined with a 3D scaffold can better promote wound healing and reduce scarring (Figure 2). All 3D scaffolds based on a single-component scaffold adopt the form of the composite and exhibit better physical properties, such as spinnability and tensile strength, as well as biological properties, such as low skin toxicity.

#### Eye

Haghighat et al. (2020) reported that the use of β-carotene as a differentiation medium and an alginate-based scaffold to induce the differentiation of ciliary epithelium-derived MSCs into advanced retinal cells. Holan et al. (2015) reported that the combination of MSCs and a nanofiber scaffold improved healing via enhanced corneal thickness, re-epithelialization, and blood vessel formation, while inhibiting local inflammation. Besides, Yazdani et al. (2019) reported that the use of an HA hydrogel crosslinked with PEG diacrylate. For ocular restoration, the optimized HA scaffold improved the morphology of sheets of oral mucosal epithelial cells, cell metabolism, and the expression of genes associated with adherence and stemness, while reducing cellular damage. M’Barek et al. (2017) reported that an amniotic membrane scaffold produced from ESC-derived RPE cells on amniotic membrane sheets. As compared to the injection of ESC-RPE cells in suspension, transplantation of sheets of ESC-RPE cells prevented the death of photoreceptor cells and increased vision despite retinal degeneration.

In summary, SC therapy combined with polysaccharide-based 3D scaffolds significantly improved eye reconstruction.

#### Nerve System

Li et al. (2018a) reported that the use of an HA scaffold modified with adhesive peptides for the treatment of SCI. MSCs with recombinant brain-derived neurotrophic factor exhibited improved cell survival and sustained gene expression *in vitro*. MSCs also effectively improved the integrity of spinal tissue, alleviated inflammation, and inhibited glial scar formation.

Caron et al. (2016) reported that a new agarose/carbomer-based hydrogel optimized the viability, density, and delivery of paracrine factors of MSCs. In a mouse model of SCI, MSCs combined with a hydrogel scaffold greatly modulated the pro-inflammatory environment, improved the numbers of M2 macrophages, and facilitated regeneration of the original environment (Shevela et al., 2019). Kim et al. (2016) reported that as compared with intralesional injection, transplantation of scaffold-based MSCs achieved a greater implantation success rate and locomotor ability in acute SCI, especially with the use of a chitosan scaffold, followed by a PLGA scaffold. Besides, Wang et al. (2020b) reported that Matrigel efficiently supported the survival and differentiation of NSCs. As compared with rats treated with phosphate-buffered saline (sham control) or with Matrigel transplants, NSCs in Matrigel improved the behavioral recovery and the expression levels of neuronal and reactive astrocyte marker in a rat model of SCI. Zweckberger et al. (2016) reported that SAP-treated animals had more living NPCs and the differentiation capability of oligodendrocytes and neurons had increased. The combined treatment with SAP and NPCs improved the reservation and behavioral outcomes of the corticospinal tract. Qing et al. (2018) reported that a novel heterostructure scaffold composed of electrospun silk nanofibers layered on graphene paper, which had high biocompatibility and conductivity, had effectively induced oriented growth and improved differentiation of neuroblastoma cells.

For stroke treatment, Moshayedi et al. (2016) reported that as a platform to promote the adhesion of structural motifs and release of growth factors, an HA-based self-polymerizing hydrogel promoted differential maturation of NPCs in the stroke cavity and improved the survival rate of NPCs, which can be tracked by magnetic resonance imaging (Guo et al., 2019; Wang et al., 2020).

For glioblastoma, Sheets et al. (2020) reported that gelatin matrices (GEMs) significantly supported the viability, persistence, and efficacy of seeded NSCs. Delivery with a scaffold of GEMs enabled therapeutic cells to persist in an immunologically active post-surgical environment, while maintaining stemness and the ability to target tumors. In a mouse model, GEM-NSCs significantly reduced the residual tumor volume. Moore et al. (2020) designed composite gelatin-electrospun scaffolds with two degradation profiles due to different ratios of cyclic to acyclic acetals (fast and slow). NSC implantation efficiency, persistence, and long-term survival were all improved by the fast and slow degrading scaffolds, respectively, and scaffold degradation had little effect on the persistence of NSCs.

**FIGURE 3:**
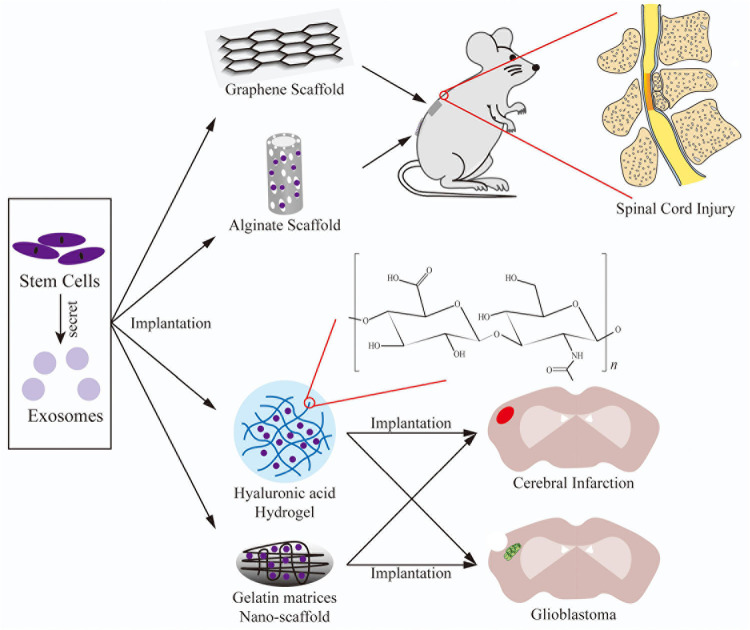
The application of 3D scaffolds combined with SCs/exosomes implanted into models of SCI, cerebral infarction, and glioblastoma formation.

This section described 3D scaffold-based SC therapy for treatment of neurological diseases, including SCI, stroke, and glioblastoma (Figure 3). SC therapy with MSCs or NSCs is advantageous for nerve repair by maintaining the survival of implanted SCs and enhancing neuron proliferation and differentiation.

### Exosome Therapy

Exosomes are produced by various cell types, including MSCs (Lai et al., 2010), NSCs, CPCs, Schwann cells (Fevrier et al., 2004), B cells (Raposo et al., 1996), T cells (Peters et al., 1989), dendritic cells (Zitvogel et al., 1998), mast cells (Zitvogel et al., 1998), tumor cells (Wolfers et al., 2001), and sperms (Sullivan et al., 2005). Additionally, in most studies, MSCs were the major source of exosomes. MSCs are simple to culture, grow quickly, and have a high capacity for exosome production (Lindroos et al., 2011; Yeo et al., 2013). MSCs are also easily isolated and manipulated, with high potential for proliferation and differentiation (Mirsaidi et al., 2012). MSC-derived exosomes have properties of donor cells, which can facilitate cellular self-repair, retain homeostasis of the microenvironment, and boost healing of injured tissues (Lai et al., 2015). In contrast to MSCs, exosomes have greater biological impacts when directly fused with the target cell. Since the active components are shielded from destruction by the plasma membrane, exosomes can be preserved and transported at low temperatures. In addition, manual monitoring of the dose, direction, concentration, and time of usage is relatively simple. Most importantly, cell transplantation poses no risk of immunological rejection or tumorigenesis (Lu et al., 2017).

### Applications of Exosome Therapy

#### Bone

Zhang et al. (2018a) reported that MSC-derived exosomes are able to repair osteochondral defects via an organized multi-faceted response that includes increased migration, proliferation, and matrix synthesis, decreased apoptosis, and modulated immunoreaction. Cui et al. (2016) reported that exosomes derived from mineralized osteoblasts significantly influenced miRNA profiles in recipient bone marrow cells, thereby promoting differentiation into osteoblasts. Axin1 expression was inhibited by changes in miRNA profiles, while β-catenin expression was increased and activated the Wnt signaling pathway.

#### Heart

Arslan et al. (2013) reported that the administration of MSC-derived exosomes decreased the infarct size and increased cardiac function in a mouse model of myocardial ischemia-reperfusion injury. After revascularization, exosome treatment greatly decreased neutrophil and macrophage infiltration, implying that exosomes have an anti-inflammation effect. Bian et al. (2014) found that hypoxic BMSC-derived exosomes were fully accepted by umbilical vein endothelial cells, resulting in increased proliferation and migration. Exosome administration decreased infarct duration, restored cardiac activity, and induced angiogenesis in the infarcted area of a rat model of acute MI. Yu et al. (2015) reported that BMSC-derived exosomes overexpressing the transcription factor GATA-binding protein 4 released more miRNA-19a. In a rat model of acute MI, exosome administration conveyed an anti-apoptosis effect under hypoxic conditions *in vitro*, while restoring cardiac activity and reducing infarct volume. MiR-19a was shown to be active in exosome cardioprotection by downregulating phosphatase and tensin homolog expression and activating the AKT signaling pathway.

#### Skin

Exosomes are becoming more widely used in regenerative medicine due to anti-inflammatory properties and the ability to promote angiogenesis through proliferative and migratory phenotypes, wound healing, and anti-aging properties.

Exosomes facilitate a major change of the recipient macrophages to the anti-inflammatory phenotype during inflammation (Regenerative et al., 2017). Exosomes have the potential to inhibit the immune response by regulating the recruitment, differentiation, and proliferation of B lymphocyte, as well as converting activated T lymphocytes to the T-regulatory phenotype (Nosbaum et al., 2015; Monguió-Tortajada et al., 2017). According to numerous reports, exosomes also have immunomodulatory effects through particular miRNAs, such as miRNA-21, miRNA-181c, and miRNA-146a (Ti et al., 2015, 2016), and various receptor signaling pathways (Li et al., 2016).

Exosomes, which promote neoangiogenesis, tissue regeneration, collagen deposition, re-epithelialization, and wound healing during proliferation (Midwood et al., 2004), can be preserved and transport their RNA and protein cargos to recipient cells to regulate migration and proliferation. Exosomes can also promote the synthesis and expression levels of types I and III collagen and elastin during the remodeling process (Zhang et al., 2015). In addition, exosomes suppress scar formation by regulating collagen production at various stages of the wound healing process (Kou et al., 2018).

#### Eye

Yu et al. (2016) investigated that intravitreal administration of MSC-derived exosomes inhibited inflammation, limited the extent of damage, reduced apoptosis, and improved visual function via downregulation of MCP-1. Zhang et al. (2018b) concluded that injection of MSC-derived exosomes into the eye after a normal pars plana vitrectomy could increase the anatomical and functional effects of macular holes, which is difficult to treat during the initial surgery. Some studies Trichonas et al. (2010); Nakazawa et al. (2011), and Xie et al. (2017) have found that MSC-derived exosomes induce the production of inflammatory cytokines and increase autophagy, thereby increasing the survival of photoreceptors. In a rat model of retinal detachment, exosomes were reported to inhibit the induction of TNF-α and subsequently suppress the inflammatory response and cell death following retinal damage by decreasing autophagy.

#### Nerve System

Xin et al. (2013) found that co-administration of BMSC-derived exosomes significantly boosted neurological regeneration and induced neurogenesis and angiogenesis in the ischemic zone after ligating the middle cerebral artery in a rat model of stroke. Doeppner et al. (2015) reported that following ligation of the middle cerebral artery, administration of BMSCs and BMSC-derived exosomes improved neurological function and stimulated angiogenesis and neurogenesis to the same degree in a mouse model of focal cerebral ischemia.

### Scaffold-Based Exosome Therapy

Although the efficacy of exosome therapy has been investigated in various systems for many years and some results have been achieved, 3D scaffold-based exosome therapy is still in its infancy, as the effects in some systems have not yet been studied. Nonetheless, the use of 3D scaffold-based exosome therapies for regeneration of bone, skin, and neurons have reportedly achieved good results.

#### Bone

In recent years, 3D scaffold-based exosome therapy has shown great potential in the regeneration of bone cartilage (Figure 1). Narayanan et al. (2016) reported that osteogenic MSC-derived exosomes can be combined with ECM proteins, such as collagen I and fibronectin, to promote the differentiation of MSCs into osteocytes. Yang et al. (2020b) reported that the combination of UCMSC-derived exosomes and HAP-embedded crosslinked HA-alginate hydrogel *in situ* significantly enhanced bone regeneration of preosteoblasts. Several studies Qi et al. (2016); Zhang et al. (2016), and Ying et al. (2020) reported that the combination of iPSC- or MSC-derived exosomes and a β-TCP scaffold promoted angiogenesis and osteogenesis. Three studies Sanchez et al. (2020); Wang et al. (2020d), and Zha et al. (2021) reported that the combination of exosomes and a PCL scaffold significantly enhanced osteogenic differentiation of MSCs. Gandolfi et al. (2020) reported that the mineral-doped PLA porous scaffolds enriched with MSC-derived exosomes increased the osteogenic commitment of MSCs. Li et al. (2018b) and Swanson et al. (2020) reported that different scaffolds based on biodegradable PLGA, in which exosomes facilitated osteogenic differentiation, promoted mineralization by recruiting endogenous cells to bone defects.

Many types of 3D scaffolds can promote osteogenesis. With the exception of common gels, polysaccharides and related complexes, some scaffolds are composed of metals, decellularized ECM, and or related complexes. Scaffolds of different materials can support the survival of SCs and promote osteogenic differentiation and osteogenesis. The combination of 3D scaffolds and exosomes provides various novel treatment methods for orthopedic injuries and promotes clinical transformation.

#### Skin

Several studies have investigated the efficacy of 3D scaffold-based exosome therapy for skin regeneration (Figure 2). Yang et al. (2020a) reported that the combination of a Pluronic F-127 hydrogel and UCMSC-derived exosomes significantly accelerated wound closure, enhanced regeneration of granulation tissue, and promoted wound healing for diabetics. Wang et al. (2020a) confirmed that a biocompatible 3D porous self-healing methylcellulose-chitosan hydrogel loaded with placental MSC-derived exosomes facilitated wound healing by synergistically inducing angiogenesis and inhibiting apoptosis. Shafei et al. (2020) reported that a alginate-based hydrogel loaded with ASC-derived exosomes improved wound closing, vessel development, and collagen synthesis. Nooshabadi et al. (2020) reported that a chitosan hydrogel scaffold containing exosomes was effective for wound closure and promoted a high degree of re-epithelialization.

#### Nerve System

Several studies have assessed the effectiveness of 3D scaffold-based exosome therapy for treatment of nerve injuries (Figure 3). Li et al. (2020) reported that topical transplantation of MSC-derived exosomes fixed in a peptide-modified hydrogel provided exosome-encapsulated ECM for repair of damaged nerve tissues and induced comprehensive mitigation of the microenvironment. The implanted exosomes exhibited better retention and continuous release. By mitigating oxidation and inflammation, the exosome-loaded hydrogel significantly elicited nerve repair. Hsu et al. (2020) reported that UCMSC-derived exosomes induced neurite outgrowth and protected neurons from formic acid *in vitro*. *In vivo*, an alginate scaffold with exosomes exhibited anti-toxicity, anti-inflammation, and pro-neurotrophy activities in a ligation model of spinal nerve pain.

## Conclusion

The SC therapy has become a fascinating biomedical field that has prompted a lot of excitement in recent years. Local injection of ASCs effectively alleviated OA of the knee (Zhou et al., 2018); iPSCs had maximum capacity in myocardial repair and iPSC-derived cardiomyocytes exhibit much of the same features as cardiac cells (Xi et al., 2010). Treatment with the combination of SAP and NPCs promoted the reservation and behavioral outcomes of the corticospinal tract (Zweckberger et al., 2016). As a potential medical option, the use of SC therapy still faces numerous challenges, including immunological rejection, teratoma formation, and differentiation of non-targeted cells.

Exosomes, which carry different bioactive proteins, nucleic acids, microRNAs, and unique gene products (Jo et al., 2014; Katare et al., 2014; Lee et al., 2015a), have long been recognized as vital for the clinical efficacy of endogenous and grafted cells (Lai et al., 2015; Zhang et al., 2018a). Exosomes can also serve as intercellular signaling mediators and transport trophic factors to neighboring cells (Colombo et al., 2014). As compared to SC therapy, the use of exosomes is minimally invasive in acellular regenerative medicine. MSC-derived exosomes can repair osteochondral defects via an organized, multi-faceted response (Zhang et al., 2018a); MSC-derived exosomes were shown to decrease the infarcted area and increase cardiac function in a mouse model of myocardial ischemia-reperfusion injury (Arslan et al., 2013); BMSC-derived exosomes significantly boosted neurological regeneration and induced angiogenesis and neurogenesis of the ischemic zone after ligating the middle cerebral artery (Xin et al., 2013; Doeppner et al., 2015).

Various biomaterials used to create 3D scaffolds have revolutionized tissue and organ transplantation and regenerative medicine. Recent advances in 3D scaffold-based therapies in different tissues are outlined in this study. The hydrogel particle scaffolds are usually suitable for various tissues and organs, while solid scaffolds are generally used as models of orthopedic diseases. The combination of iPSC- or MSC-derived exosomes and β-TCP scaffolds promote angiogenesis and osteogenesis (Qi et al., 2016; Zhang et al., 2016; Ying et al., 2020); The combination of Wharton jelly-derived MSCs with PEG, HA, and chitosan improved defect size and cardiac function, while promoting neoangiogenesis and cardiomyogenesis (Rabbani et al., 2017); MSC-derived exosomes fixed in a peptide-modified hydrogel significantly elicited nerve recovery by mitigating oxidation and inflammation (Li et al., 2020).

As carriers, 3D scaffolds combined with SC/exosomes provide a 3D microenvironment and played a role in continuous infiltration and release. The combination of a 3D scaffold, especially with exosomes, can better improve treatment of bone and cartilage defects, myocardial repair, and nerve repair more than normal SC/exosome therapies. In the future, 3D scaffold-based SC/exosome therapy will be applied for successful treatment of different tissues. Particularly, 3D scaffold-based exosome therapy presents much stronger advantages without the limitation of SCs. The combination a 3D scaffold and SCs will continue to advance from fundamental research to clinical application. 3D scaffold-based exosome therapy is expected to become more widely applied in the future.

## Author Contributions

CG and JF contributed to writing the manuscript and conceive the outlook of figures. AW and YD contributed to writing the manuscript. YZ, JL, and WC contributed to finish the figures. SH and LC contributed to launche the theme. All authors contributed to the article and approved the submitted version.

## Conflict of Interest

The authors declare that the research was conducted in the absence of any commercial or financial relationships that could be construed as a potential conflict of interest.

## Publisher’s Note

All claims expressed in this article are solely those of the authors and do not necessarily represent those of their affiliated organizations, or those of the publisher, the editors and the reviewers. Any product that may be evaluated in this article, or claim that may be made by its manufacturer, is not guaranteed or endorsed by the publisher.

## Abbreviations

SCs: stem cells
3D: three-dimensional
HA: hyaluronic acid
PCL: poly caprolactone
PDMS: polydimethylsiloxane
PEO: polyethylene oxide
PLA: polylactic acid
PLGA: poly (lactic acid-glycolic acid)
PVA: polyvinyl alcohol
PEG: polyethylene glycol
TCP: tricalcium phosphate
ESCs: embryonic stem cells
iPSCs: induced pluripotent stem cells
MSC: mesenchymal stem cell
BMSC: bone marrow MSC
ASC: adipose MSC
UCMSC: umbilical cord MSC
NSCs: neural stem cells
NPCs: neural progenitor cells
OA: osteoarthritis
MI: myocardial infarction
RPE: retinal pigment epithelium
SAP: self-assembling peptide
RADA: self-assembling gel-forming core sequence
ECM: extracellular matrix
iVPCs: induced vascular progenitor cells
GEM: gelatin matrices.
